# General Instructions for Using the IntelliCage with Mice

**DOI:** 10.1002/cpz1.70314

**Published:** 2026-01-30

**Authors:** Pia Kahnau

**Affiliations:** ^1^ German Federal Institute for Risk Assessment (BfR) German Centre for the Protection of Laboratory Animals (Bf3R) Berlin Germany

**Keywords:** cognition, home cage based, IntelliCage, learning, mice

## Abstract

The IntelliCage is a home‐cage‐based radiofrequency identification (RFID) test system for studying learning behavior of group‐housed mice. Specifically, the mice must learn where and how to get water within the IntelliCage. The protocol describes how to set up the IntelliCage, how to set up and start a learning experiment, what to consider before starting an experiment, and how to clean the system. © 2026 The Author(s). *Current Protocols* published by Wiley Periodicals LLC.

**Basic Protocol 1**: General instructions for using the IntelliCage with mice

**Basic Protocol 2**: IntelliCage cleaning

## INTRODUCTION

**Figure 1:**
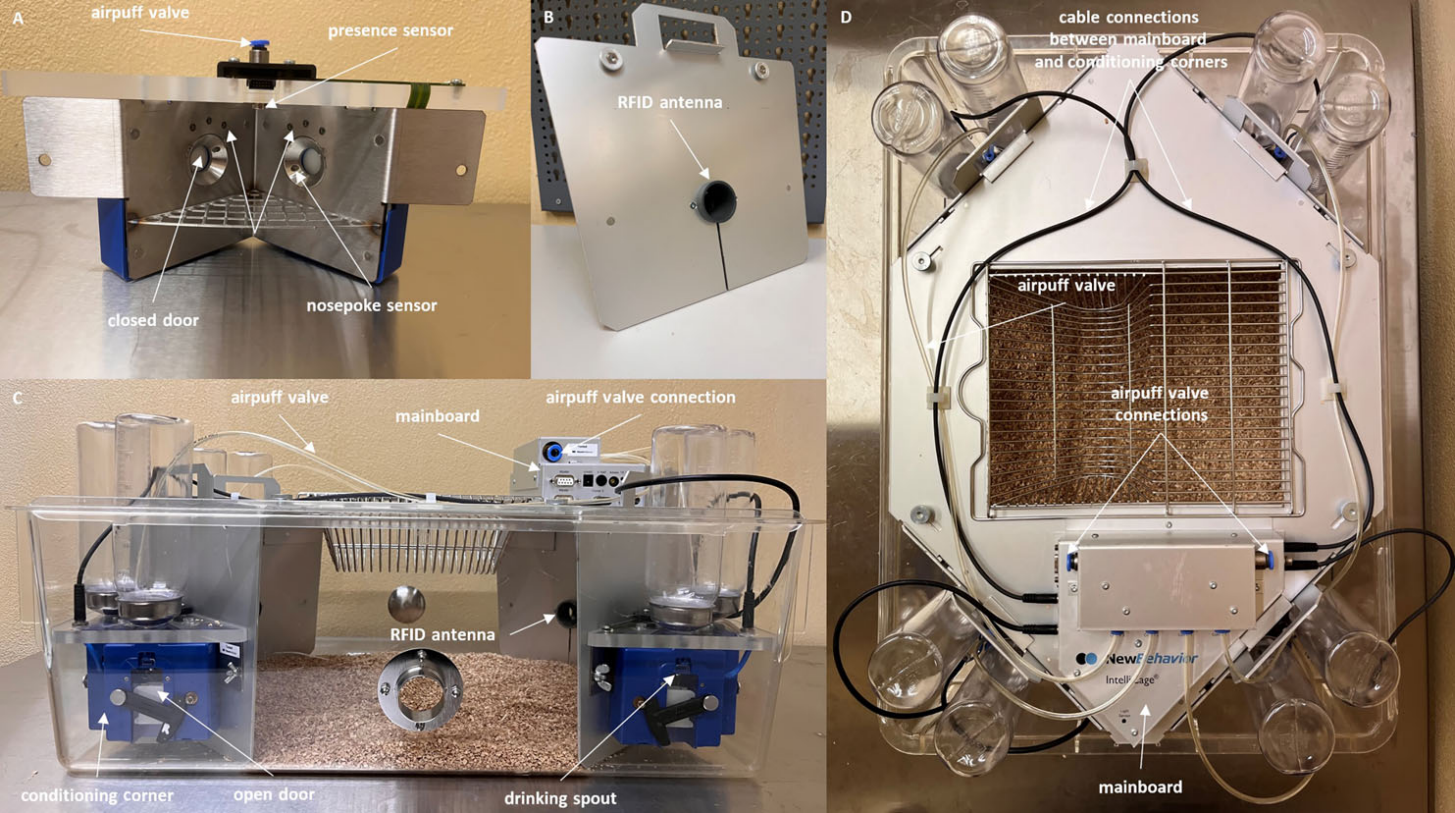
IntelliCage. (**A**) Interior view of a conditioning corner with the outer wall (**B**) removed. Inside is a presence sensor (heat sensor) that detects temperature changes. Each time a mouse enters the conditioning corner, this (together with an appropriate signal from the RFID antenna) is registered as a visit. The RFID antenna and presence sensor must be active together, ensuring that no false visits are registered (such as instances in which a mouse passes by the RFID antenna outside the conditioning corner). The conditioning corner contains LEDs and nosepoke sensors (infrared sensors). The IntelliCage can be configured to open the doors only when the mice perform a nosepoke on the sensor. (**B**) Wall of the conditioning corner, in which an RFID ring antenna serves as the entrance to the conditioning corner. Each time the mouse enters the corner, the RFID transponder located in the mouse's neck region is read. (**C**) Side view of the IntelliCage. Two conditioning corners can be seen, with their doors open and drinking spouts visible. In this state, the mice would have the opportunity to drink water within the conditioning corner. (**D**) View from above of the IntelliCage, showing all eight water bottles (two per conditioning corner), several cables, and the mainboard.

The IntelliCage (NewBehavior, TSE GmbH, Germany) is a home‐cage‐based test system for mice (or rats) that can be used to study learning and memory behavior in social groups. Four conditioning corners, which are connected to a computer or laptop via a mainboard, are located in a Tecniplast P2000 (type IV) cage (Fig. 1). Each conditioning corner has space for only one adult mouse. However, if the mice are very young (e.g., 4 weeks old), more than one can fit inside a conditioning corner. This should be taken into account when planning experiments.

A **R**adio**F**requency **ID**entification (RFID) ring antenna serves as the entrance to the conditioning corner. When a mouse enters a conditioning corner, its RFID transponder is detected, and together with activation of the presence sensor, this is registered as a visit. Water is only available inside the corners (two per corner), and access to the water can be controlled via doors. For example, the doors will only open if the mouse makes nosepokes at the nosepoke sensors inside the corner, allowing it to reach the drinking spout. The number of licks is recorded using a lickometer. To enable further stimuli to be provided, LEDs are located in the corners. As a punishment, airpuffs (e.g., 0.5 bar) can be emitted each time a mouse performs a nosepoke on the incorrect nosepoke sensor (Fig. 1A and B). The system also records the number of nosepokes made.

Different experiments can be performed using different design parameters. Mice have to learn in which corner, on which side, or in which order they have access to water at what time. A relatively simple learning experiment in the IntelliCage is “Place Learning.” Here, each mouse is assigned one of the four corners as its individual correct corner, such that the mouse can open the doors with a nosepoke to get access to the water only in that corner—all the other doors in all other corners remain closed. This relatively simple learning experiment can be used to investigate spatial short‐term memory (Endo et al., 2011; Kahnau et al., 2021; Kiryk et al., 2020; Krackow et al., 2010). An airpuff can be used as a punishment—for example, to investigate avoidance behavior (Voikar et al., 2010). It is also possible to set the system to require a certain number of nosepokes to open the doors (Kahnau et al., 2022a, 2022b) or to play sounds when a mouse enters a corner (Kahnau et al., 2023).

RFID technology enables individual data to be collected from group‐housed mice. Depending on their body size, up to 16 mice can be housed in one IntelliCage. However, it should be noted that the more mice in a cage, the less enrichment space is available. To provide more space, another cage can be connected to the IntelliCage via a simple tube. In addition, individual settings can be configured for each mouse in the group. However, care must be taken when setting up the experiment to ensure that it is not so complex as to prevent the mice from having a fair chance to learn and thus gain access to water (Kahnau et al., 2021).

The IntelliCage allows individual data to be obtained from mice kept in groups, and enables the animals to be tested 24/7 over several days, weeks, or even months. As the IntelliCage serves as both a home and test cage, the animals can be tested in their familiar environment during their active phase (the dark phase). This eliminates the need to adjust the light cycle, as is practiced in some facilities. Additionally, handling of the animals can be minimized, as they do not need to be transferred to special testing equipment for habituation or testing. Depending on the experimental design and test, this may be necessary several times a day over a period of weeks. The animals also do not need to be separated from their group members during the testing phase. Separation, an unfamiliar environment, and handling itself can all negatively impact the well‐being of mice (Gouveia & Hurst, 2013; Hurst & West, 2010; Krohn et al., 2006; Manouze et al., 2019; van Bogaert et al., 2006). Testing within the home cage also saves time, as scientists do not need to be present during the testing phase, thereby reducing their influence on the animals. This, in turn, can improve the reproducibility of the data. The learning behavior of different mouse strains in the IntelliCage has been investigated, with the results being compared across different laboratories. These results showed that existing differences between mouse strains were consistent across laboratories, with no interactions detected between laboratory and mouse strain (Endo et al., 2011; Krackow et al., 2010).

These protocols describe how to set up the IntelliCage, how to set up and start a learning experiment, and what to consider before starting an experiment (Basic Protocol 1), as well as how to clean the system (Basic Protocol 2). This article does not explain how to design or carry out a particular learning experiment or a new method. Instead, it focuses on the general use of the IntelliCage. It primarily describes useful features that are not covered in the supplied manual; some information could only be obtained through personal experience, and certain challenges only arose during the experiments themselves.

*CAUTION*: All animal experiments must be approved by the appropriate authorities and may only be carried out in accordance with the Animal Welfare Act.

## STRATEGIC PLANNING

Before starting any experiment, it is necessary to consider the procedure. The same applies to experiments with the IntelliCage (Table 1). First, it is advisable to familiarize yourself with the literature and the learning experiment designs described therein. It is also important to exchange knowledge and ideas with scientists who have experience with the IntelliCage. Such exchanges can take place via the Behavior Forum (https://www.thebehaviourforum.org/), which was developed by the EU COST Action TEATIME (TEATIME: Improving Biomedical Research by Automated Behavior Monitoring in the Animal Home‐Cage). The forum facilitates scientific exchange. You can ask your own questions or search for answers in existing threads.

**Table 1:** Checklist for Planning and Conducting an IntelliCage Experiment

Phase	To do
Planning an IntelliCage Experiment	Literature research and exchange with the scientific community, e.g., in https://www.thebehaviourforum.org. Determine how many animals have to be tested and how many IntelliCages are available. Include refinement strategies. Define the minimum number of licks that the mice must perform each day to avoid having to give them water separately, outside the IntelliCage. Define the learning criterion at which value mice must be excluded from the experiment.
Obtaining authorization for animal experiments	Comply with applicable country regulations.
Designing the IntelliCage experiment	In order to enable real change in learning, avoid overly complex designs. Include a habituation phase. Include cleaning phases and note that cleaning has an effect on mouse activity. Depending on the experimental design, breaks between individual experiments may be useful/necessary.
Preregistration	e.g., at https://www.animalstudyregistry.org.
Arrival of the mice	Allow for acclimatization time, e.g., 2 weeks. Perform transponder implantation. Perform handling training, e.g., tunnel handling (see https://wiki.norecopa.no/index.php/Mouse_handling).
IntelliCage phase	Check technique, as described in Basic Protocol 1, steps 1‐214 (i.e., check all cables, connections, sensors, LEDs, doors; check all sensors; check that LEDs turn on and off; check that doors open and close; check lickometer function). Habituate mice to the IntelliCage. Perform IntelliCage experiments: • Consider whether breaks between individual experiments are useful/possible; • Cleaning plan: consider when IntelliCage should/must be cleaned. Perform data backup.
End of IntelliCage phase	The final cleaning should be carried out as soon as the IntelliCage phase is complete, as any contamination could damage the technology long term.

In advance, it should be clarified how many animals need to be tested to answer the scientific question, whether enough IntelliCages are available, and how the process can/must be organized with different batches.

Additionally, consideration must be given to how often and when the cages need to be cleaned. This depends on how many animals are kept together as a social group and whether they have additional space in the form of an extra connected cage (e.g., via a simple tube).

If more space is available, it is also possible to provide the animals with additional stimuli in the form of various shelters, climbing opportunities, or—if the experimental design allows—a running wheel. Environmental enrichment contributes to animal well‐being and can positively affect the data (Hobbiesiefken et al., 2023).

Because males mark significantly more than females, more frequent cleaning may be necessary when working with male mice. Cleaning is primarily necessary to prevent contamination‐related damage to the equipment, but also for hygienic reasons. However, it should be noted that cleaning can influence the behavior of the animals (Pernold et al., 2019), and therefore it should not be carried out during an ongoing learning phase.

Once all considerations regarding the procedure have been finalized, it is advisable to pre‐register the study. Pre‐registration at a platform such as https://www.animalstudyregistry.org/ promotes transparency and traceability, and helps to ensure that studies are not unnecessarily repeated. This is entirely in line with the 3R (replace, reduce, refine) principle for animal research, as it reduces the number of laboratory animals required.

If animals have been ordered from a breeder, they should be given ∼2 weeks to acclimatize before experiments. If the mice were housed individually or in small groups before the IntelliCage experiment, they should be housed together for at least 1 week beforehand. This will enable them to form a reasonably stable social group. Particular attention should be paid to aggressive behavior in males.

Anywhere from 1 week to 2 days before the mice are placed in the IntelliCage, transponder injection should be performed under anesthesia/analgesia, as required by the regulations. The transponder should be placed subcutaneously in the neck region (∼1 cm behind the ears). If the transponder is placed too far back (at the back of the spine), as the mice get older (and therefore larger), it may be difficult for the RFID antenna in the IntelliCage corners to read the transponder number.

*NOTE*: All protocols involving animals must be reviewed and approved by the appropriate Animal Care and Use Committee and must follow regulations for the care and use of laboratory animals.

## Basic Protocol 1 GENERAL INSTRUCTIONS FOR USING THE IntelliCage WITH MICE

This protocol outlines the necessary steps for conducting an IntelliCage experiment. The individual steps, from designing a learning experiment and checking the IntelliCage before and during the experiment, to creating and starting an IntelliCage learning experiment, are described in a practical order. Information on habituating animals to the IntelliCage and cleaning it is also included. Drawing on years of experience, we have created a Troubleshooting Guide to help readers avoid unnecessary (beginner) mistakes (see the Troubleshooting section of the Commentary at the end of the article).

### Materials

**Figure 2:**
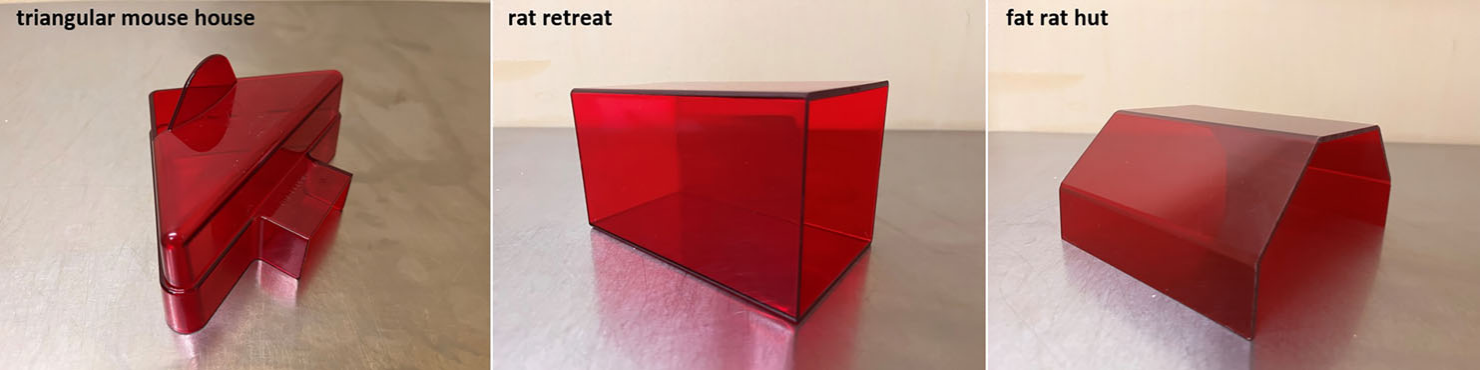
Three shelters that can be positioned under the grid lid. Note that although the supplier describes these using the word “rat”, this does not preclude their use with mice.

IntelliCage with all ports and cables supplied (e.g., RS‐232 cable)
USB‐serial adapter
Eight per IntelliCage: water bottles (scaled up to 250 ml, total volume 280 ml, Ø 56 × 154 mm, round shape with silicone seal; TSE Systems or Tecniplast) with long drinking spout (e.g., Tecniplast ACCP3521)
Small grid lid in which to offer food (if a second cage is connected for additional space, the food can also be offered in the second cage)
Computer or laptop
IntelliCage Plus Software (with three software modules: Designer, Controller, and Analyzer)
Makrolon cage type 2000P (Tecniplast)
Houses (Fig. 2), which must be positioned under the grid lid if food is only offered in the IntelliCage (this enables the mouse to reach food within the IntelliCage; the cage floor itself is too far from the feeder to allow that);
if a second cage is provided, a shelter can also be placed there
Bedding and nesting material
Tunnel, for tunnel handling (see video tutorial for mouse handling at https://wiki.norecopa.no/index.php/Mouse_handling)
Appropriate enrichment items (see Hobbiesiefken et al., 2023, for a list of suitable enrichment items; depending on available space and the enrichment, these may be positioned in an adjacent cage)
RFID transponder (e.g., Euro ID, FDX‐B, ISO 11784/85) and RFID reader

#### Design of the experiment

Experiments are designed in the Designer of IntelliCage Plus software package. This example protocol was generated using version 3.6.2.0 IntelliCage Plus (32 Bit) from TSE Systems. Minor differences between versions are possible. As an example, a Place Learning experiment for four mice is described here.

If there are more than four mice in the IntelliCage, it is advisable to distribute them across all corners as much as possible. To achieve this, determine the least preferred corner for each mouse during the habituation phase and assign it to that corner. This avoids any possible corner preference, which could have a negative impact on the results.

The mice have to learn where (place) in the IntelliCage they have access to water. Each mouse is assigned to one correct corner, and the other three corners are considered wrong. Only the doors in the correct corner can be opened by a nosepoke at the nosepoke sensors.

**Figure 3:**
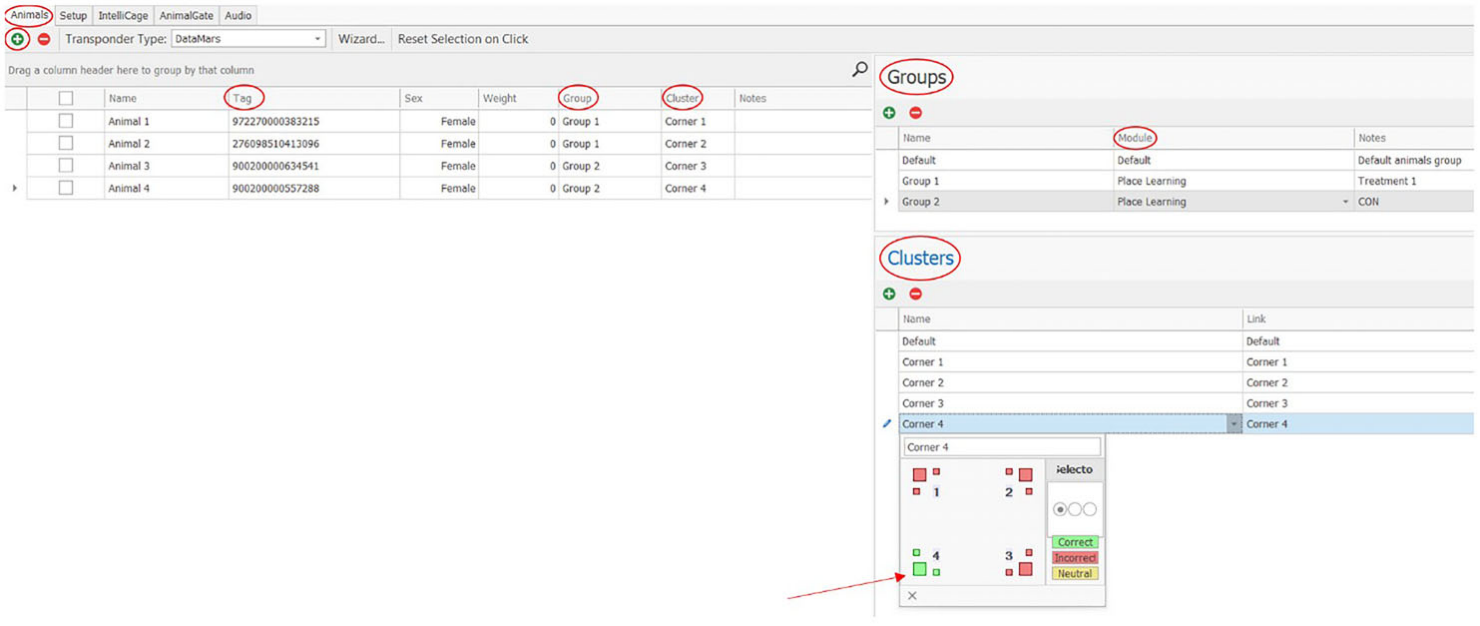
Animal tab of the Designer in the IntelliCage Plus Software.

1 Open the Designer (Fig. 3); the tabs “Animals”, “Setup”, “IntelliCage”, “AnimalGate”, and “Audio” can be seen at the top left of the screen.

**Figure 4:**
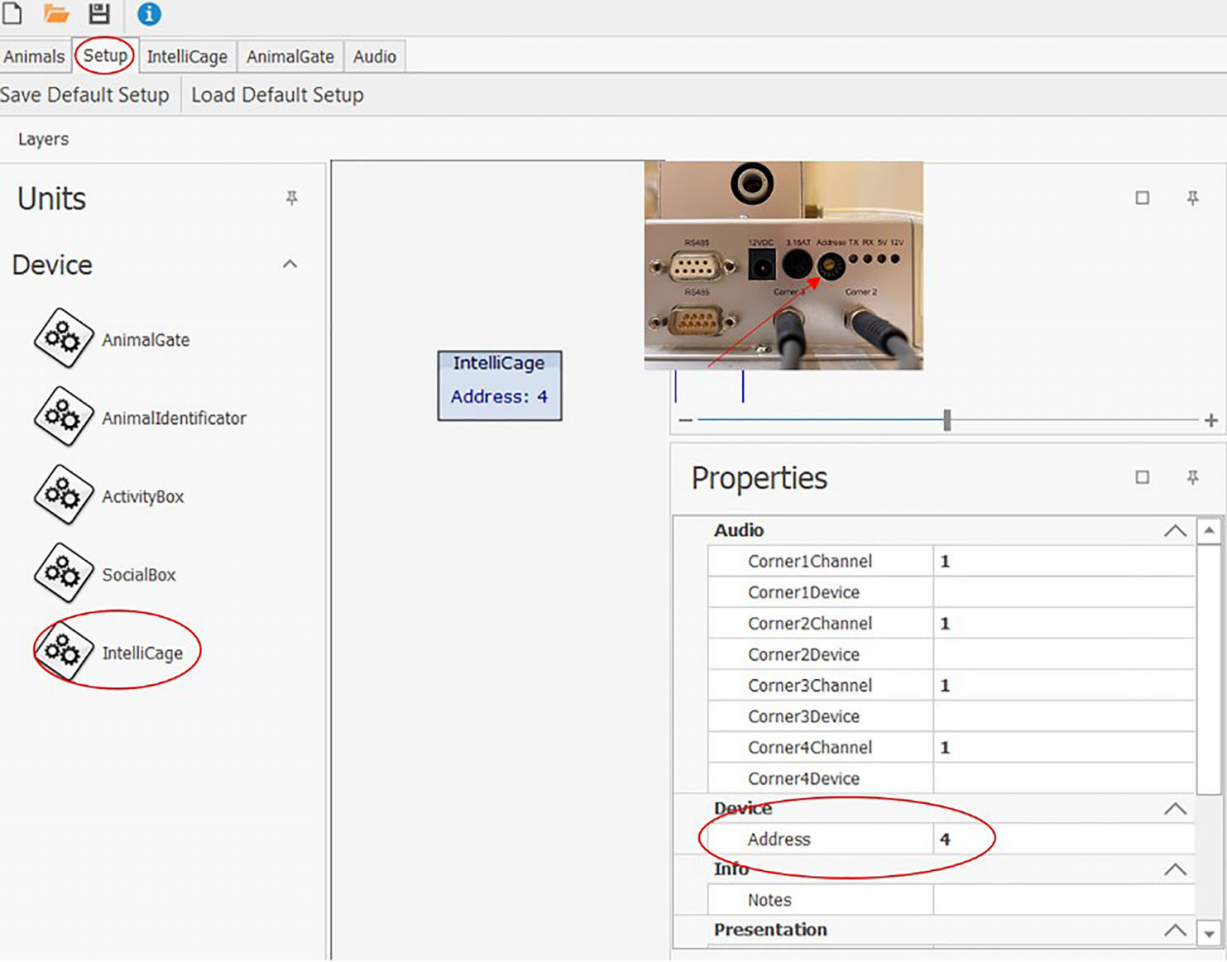
Setup tab of the Designer in the IntelliCage Plus software and where to find the address number on the IntelliCage mainboard.

2 Click on “Animals”.
  a. All animals must be added.
  b. Click the green “plus” button (top left) and enter the transponder number to “Tag”.
  When different groups (e.g., treatment and control) are tested together in one IntelliCage, the mice can be assigned according to the groups.
3 Create mouse groups under “Groups” (Fig. 3, top right).
  a. The groups can be described under “Notes”.
  b. Assign each group to a “Module”
    - Modules are created in the “IntelliCage” tab.
    - Depending on the experiment, it may not be necessary to create different modules; in this case, the same module can be assigned to all the groups.
    - The individual groups are assigned under “Group”.
4 Create clusters and assign each animal to a “Cluster”.
  a. If there are more than four animals, the correct corner will be the same for more than one animal. In this case there are four configurations with one correct and three incorrect corners. These four configurations are termed clusters. Each individual animal is assigned to one of these clusters.
  b. The clusters can be defined at the bottom right under “Cluster”. The individual corners and water bottles (represented by boxes) can be defined as “Correct” (green), “Incorrect” (red), or “Neutral” (yellow).
  c. In the Place Learning experiment, only one corner is correct (green) and all the others are incorrect (red). However, an individual corner can be assigned to each mouse.
5 Choose transponder type.
6 Click on “Setup”.
  Each individual IntelliCage must be added under the “Setup” tab (Fig. 4). You can include more than one IntelliCage in your setup.
  a. Make sure that each device has the correct device address.
  b. The address of the IntelliCage can be found on the side of the mainboard (see miniature photo in Fig. 4).

**Figure 5:**
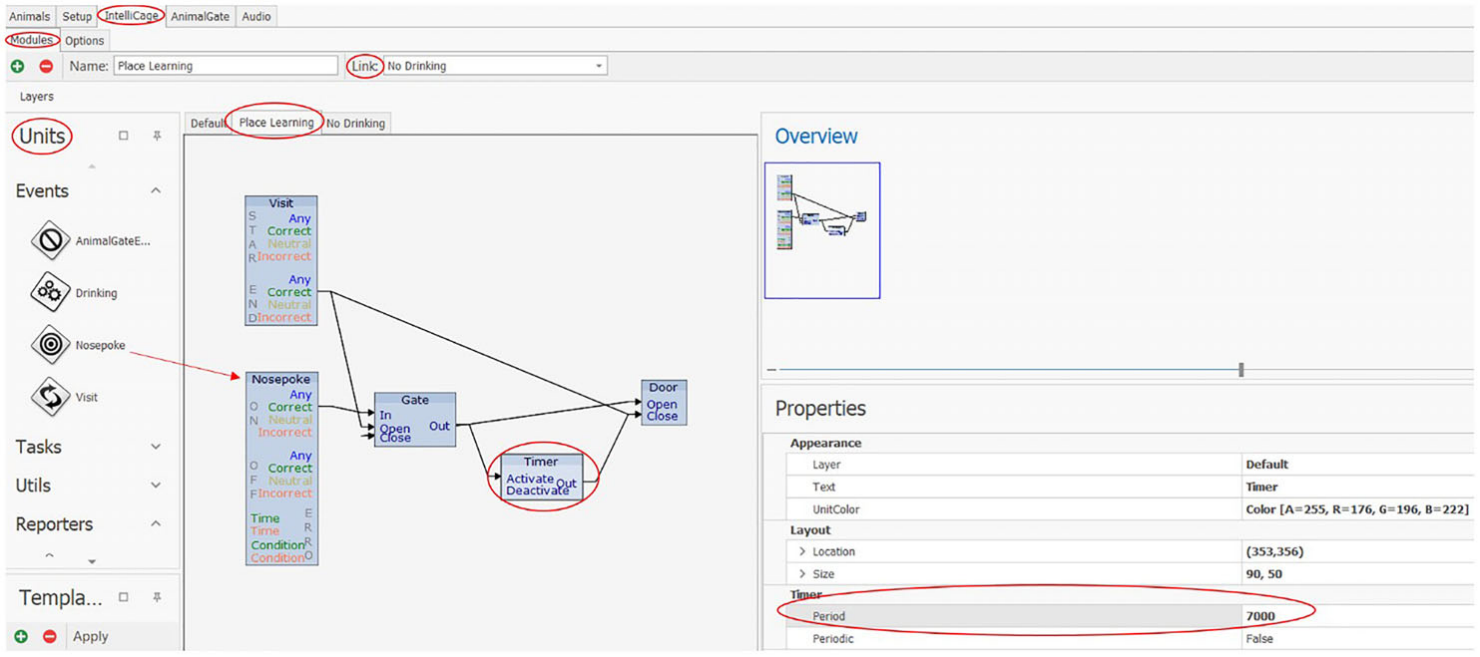
IntelliCage‐Modules tab of the Designer in the IntelliCage Plus Software.

7 Click on IntelliCage under “Units” and drag it to the left into the window.
8 Click on the IntelliCage to open a window on the right side.
  The address can be adjusted there as needed under “Address”.
  In the “IntelliCage” tab (Fig. 5), various modules can be created under “Modules”. In this tab, the conditions under which the mice can access water are defined. The creation of experiments follows the if‐then principle. During Place Learning, the mice can open the doors to the water bottles only through a nosepoke in the correct corner (assigned to each mouse); nosepokes in the incorrect corner have no effect. In this example, the doors in the correct corner should only be opened once per visit (when the mouse enters the corner, whereby the RFID antenna and the presence sensor are activated). To re‐open the doors in the correct corner, the mouse must first leave and then re‐enter it.

**Figure 6:**
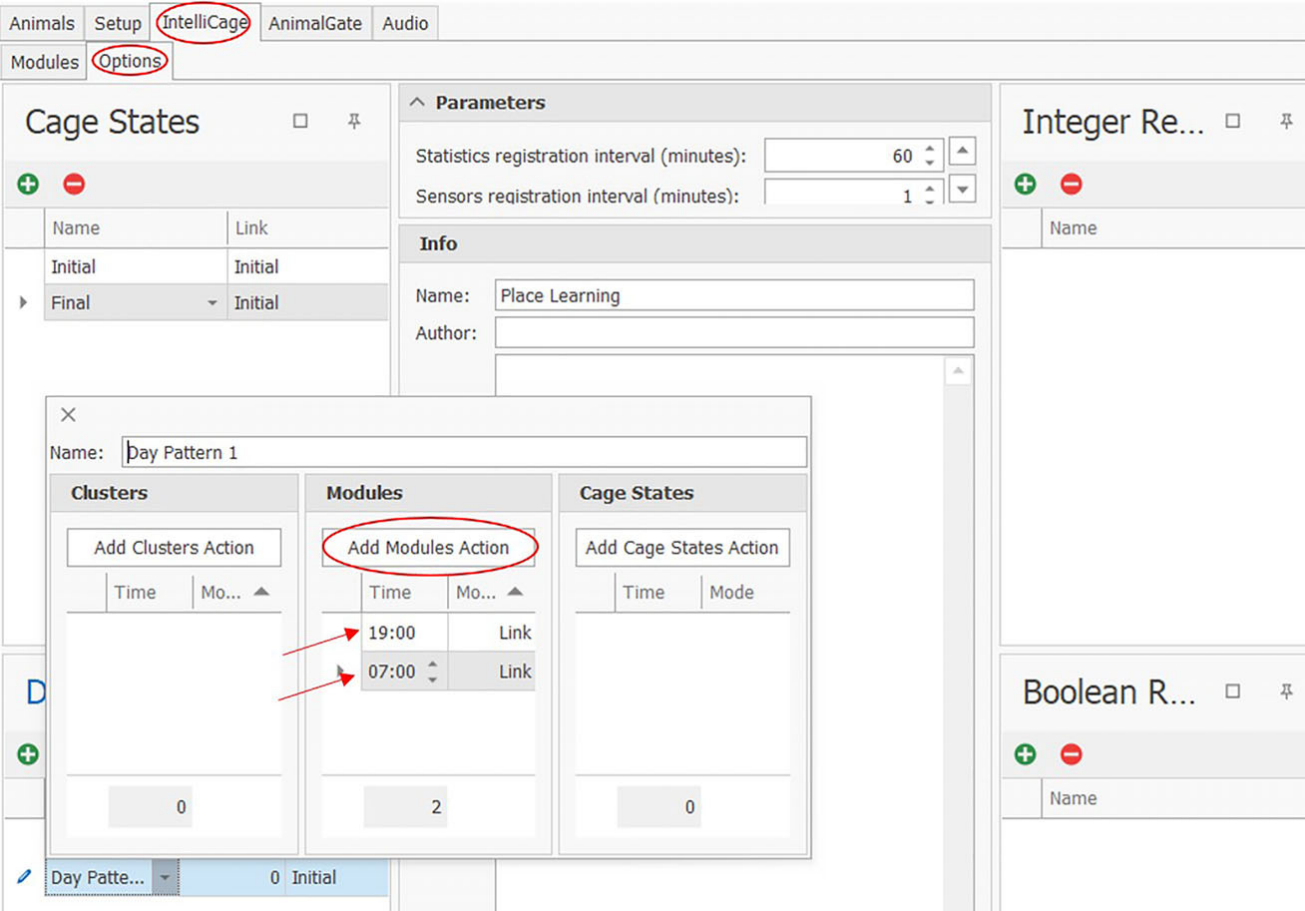
IntelliCage‐Options tab of the Designer in the IntelliCage Plus Software.

9 Click on “IntelliCage” and on “Modules”.
10 Choose the necessary items: *Visit*, *Nosepoke*, *Gate*, *Timer* and *Door*; the corresponding items are selected (via drag and drop) under “Units” (left).
11 Connect individual items as needed by first dragging and dropping the individual items into the window, and then creating connections (arrows) between two items by clicking the first item at the point where you want to start (e.g., *Nosepoke ON* “Correct”) and dragging it to the next item. Examples:
  a. An arrow from *Nosepoke ON* “Correct” to *Gate* “In” ensures that a correct nosepoke leads to a further action. A connection from *Nosepoke* ON “Any” leads to an action being performed by nosepokes in all corners.
  b. An arrow from *Gate* “Out” (representing a virtual gate within the designer software to allow more complex programming; this will become clearer as you read on) to *Door* “Open” causes the door to open when the correct nosepoke is made.
  c. An arrow from *Gate* “Out” to *Gate* “Close” creates a connection that allows the mouse to open the doors only once with a nosepoke during a visit. To open the doors again, the correct corner must first be left and re‐entered and a new nosepoke must be made.
  d. An arrow from *Gate* “Out” to *Timer* “Activate” makes it possible to keep the door open for a defined period of time; clicking on *Timer* causes a window to open on the left, where a time period can be set in milliseconds.
    How long the doors should be open depends on the experiment. Keep in mind that a short door time (e.g., 5 sec) provides less time for drinking, as the mice have to visit the correct corners more often to drink enough, but it also increases the number of trials.
  e. An arrow from *Timer* “Out” to *Door* “Close” causes the door to close after the time has elapsed. Another nosepoke while the mouse is still in the correct corner will not open the door because the *Gate* prevents the door from opening again before the mouse leaves and re‐enters the corner.
  f. An arrow from *Visit END* “Correct” to *Door* “Close” causes the door to close as soon as the mouse leaves the corner before the timer runs out. If the timer elapses when the door is already closed, this does not matter as the door will only close one time.
  g. An arrow from *Visit END* “Correct” to *Gate* “Open” ensures that the door can be opened again with a new visit and a new correct nosepoke. The Gate is now open again for new information.
  h. Further modules can be created. For the example, for a Place Learning task including drinking and non‐drinking phases, a switch between two modules is to be made at certain times. The two modules are Place Learning and No Drinking. The No Drinking module contains only the item *Nosepoke* but without any connections to other items, as no doors (not even the correct ones) should be opened during this module. It is also possible to link *Nosepoke ON* “Correct” to a reporter and *Nosepoke ON* “Incorrect” to another reporter to determine how many correct and incorrect nosepokes were made, although the doors cannot be opened during the non‐drinking phase. If the mice should only have access to water at defined times (for example, only during the dark phase, e.g., 7:00 pm to 7:00 am), these can be set under the “IntelliCage” and “Options” tabs (Fig. 6).
    - Click on “IntelliCage” and “Options”.
    - If needed, further Day Patterns can be added and set by clicking on the green cross in the Day Pattern settings, found at the bottom left:
      ○ Click on “Day Pattern 1” (a new window will open).
      ○ Click on “Add Modules Action”.
      ○ Enter desired time (in this example, a change is set to take place at 7:00 pm and at 7:00 am, between the linked modules).
      ○ Set linking under “IntelliCage” and “Modules” (see Fig. 5; Name: Place Learning, Link: No Drinking).
**After the experiment is created in the Designer module of the software, it must be saved and can be opened and started in the Controller module**. In general, if you are going to present several learning tasks in a row, you should consider whether it would be better to take a break between different learning experiments. For example, breaks can be implemented as a new module (“have_a_nice_weekend”) with all doors open in all corners. However, this depends on the research question and the complexity of the experiments.

#### Sensor control

The functionality of all sensors should be checked before starting the experiment and after each cleaning.

**Figure 7:**
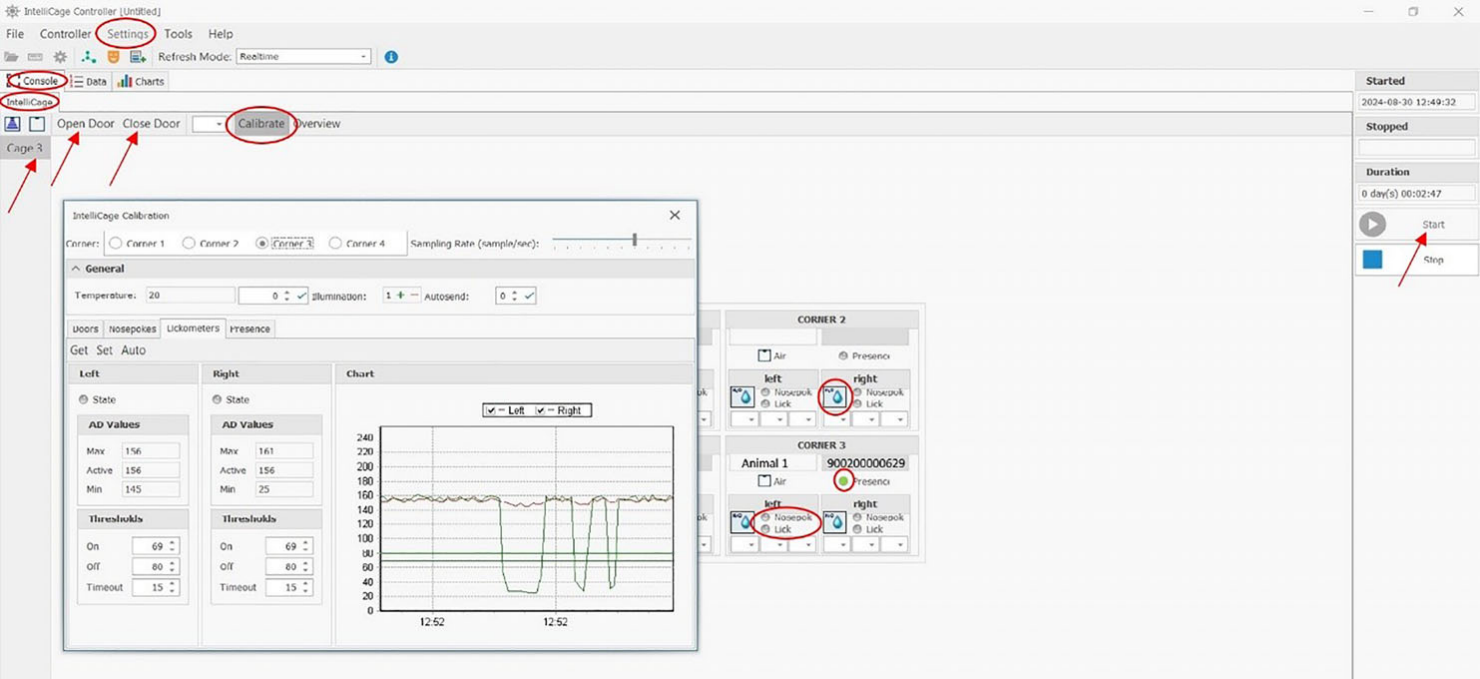
Console of the Controller in the IntelliCage Plus Software.

12 Start an experiment in the controller of the IntelliCage Plus software:
  a. At the top left, you will see the tabs “FILE,” “CONTROLLER”, and “SETTINGS”; click on “Settings” ‐> a window opens.
  b. Click on “Experiment” ‐> a window opens.
  c. Load Experiment File ‐> click on the corresponding experiment.
  d. Click “Start” (at right).
  The Controller (Fig. 7) shows, among other things, the four corners and the respective sensors. The blue water droplets indicate that the doors are open; if they appear gray, the doors are closed. If there are mice in the IntelliCage and they are currently in a corner, the Controller will show which mouse it is and whether it is nosepoking and/or drinking.

13 Check doors:
  a. Click on the “Console”.
  b. Click on the “IntelliCage”.
  c. Click on one of the cages (if more than one is used, the left side).
  d. Click on “Open Doors” and see if the doors open.
  e. Click on “Close Doors” and see if the doors close again.
    If the doors do not open and close properly, check whether there is a loose object in the door.
14 Check sensors.
  The presence sensor and RFID antenna can be tested by holding an RFID transponder in hand and placing it in the corner under the presence sensor. This should cause both the presence sensor to light up green and the corresponding transponder number to be displayed. The presence sensor may not respond if the hands are too cold. If the nosepoke sensors are touched, this should also light up green. When you touch the drinking spout, “Lick” should also light up green (only works without gloves!). Under “Calibrate”, the values and thresholds can be checked for all sensors of each corner.
  If individual sensors fail to work, it is advisable to contact TSE if you do not have the technical knowledge to repair the sensors. Note that when individual corners are exchanged, the mice avoid these corners at first, which could influence a running experiment.

#### Preparation of IntelliCage

15 Cover the cage floor with 4‐5 cm of bedding. If no second cage is connected, place the shelters under the grid lid in the middle of the cage; if a second cage is connected, the food and shelters can be placed there. Distribute nesting material in the cage.
16 Place enrichment items (e.g., the handling tube and gnawing sticks) in the cage. Further enrichment can be provided depending on the space available.
17 Fill the water bottles to a maximum of 250 ml.
  Experience shows that if there is >250 ml water in the bottle, not a single drop comes out due to underpressure caused by a near vacuum in the top of the bottle.
18 Connect the IntelliCage to the PC/laptop via standard serial cable RS‐232 cable. A USB‐serial adapter may be necessary.

#### Experimental procedure

**Figure 8:**
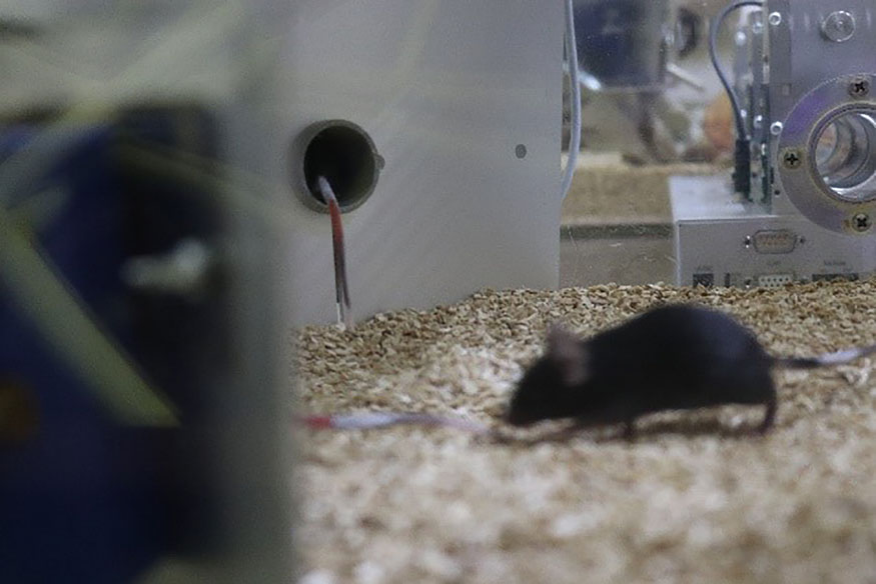
Mice with a colored mark on their tails.

19 Place all transponder‐bearing mice in the IntelliCage to start the corresponding experiments.
  To make it possible to distinguish the mice individually, they can be marked on the tail (e.g., using an Edding 750 paint marker; Fig. 8). However, this color marking must be reapplied weekly.

**Figure 9:**
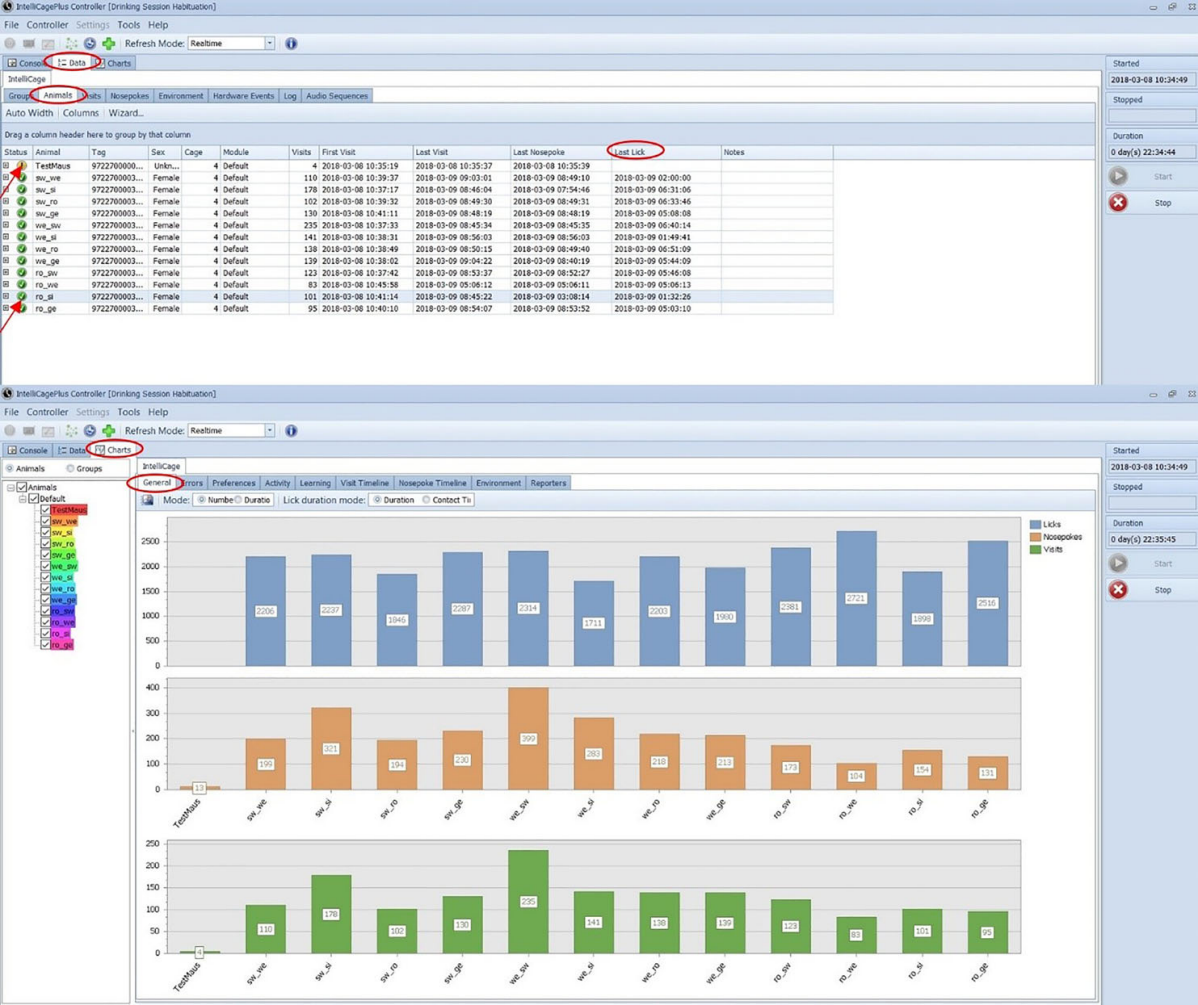
Lick‐control with the “Controller” of the IntelliCage Plus software.

20 First, habituate the mice to the IntelliCage so that they learn how to get water (with all corners set as correct for all mice), through the following steps:
  a. All Doors Open: Keep all doors are open in all corners. This habituation phase should last for at least 2 days.
    It is possible to keep the mice in the IntelliCage with all doors open before transponder implantation. Of course, data collection will not begin until the mice have had transponders injected.
  b. Visit Opens Doors: To habituate the mice to the sounds and vibrations that may occur when the corner doors open, begin with all doors closed but have doors open as soon as the mouse enters a corner, and then close again as soon as the mouse has left the corner. This habituation step should last for at least 2 days.
  a. Nosepoke Open Door: To teach the mice to open the doors with a nosepoke, begin with all doors closed but have doors open as soon as the mouse nosepokes at the nosepoke sensor in a corner. This habituation phase should last for at least 2 days.
  d. Drinking Phase Habituation: If the mice will only have access to water at certain times (e.g., two drinking periods during the dark period), habituate the mice to these drinking periods. This habituation phase should last for at least 1 week.
21 Perform daily control checks:
  a. Because the mice have to learn when and where they have access to water, it is important to check daily whether the mice have drunk any. The system gives the first warning message if no visit has taken place within 12 hr. If a mouse has not drunk within 24 hr, another warning is given. Mice that have not drunk at all or have drunk too little (based on a threshold that should be defined before starting the experiment) must be offered water separately. A cage (e.g., type II or III) can be provided for this purpose, in which the mouse is offered water and observed while drinking. Attention should also paid to other possible symptoms that could indicate stress. If the mouse drinks, behaves inconspicuously, and shows no abnormalities, it can be returned to the IntelliCage. However, if the mouse is abnormal in any way, a veterinarian should be contacted to discuss further action.
  b. A decision should be made before the experiment regarding when to exclude mice from the experiment. Repeated failure to drink indicates a lack of learning behavior. If a mouse needs to be excluded, this mouse can be given unrestricted access to the water bottles (settings in the Designer). This allows the social group to remain unchanged.
  c. The individual animals are listed under “Data”, “IntelliCage”, and “Animals” (Fig. 9); a yellow exclamation mark indicates that a mouse has not drunk water, and a green tick indicates that it has. “Last Lick” shows when the mouse last drank. If a mouse has not drunk during the last dark/active phase, it is necessary to check whether it is showing any other abnormalities, and possible consider giving it water separately.
  d. The number of visits, nosepokes, and licks can be viewed individually for each mouse under “Charts” and “General” (Fig. 9). Keep in mind that the number of visits, nosepokes, and licks is counted from the beginning of the experiment.

#### Data collection and analysis

22 Perform planned data analysis:
  a. All data recorded during the experiment—number of visits (correct and incorrect), visit time, number of nosepokes (correct and incorrect), number of licks—are stored on the PC/laptop under Documents ‐> New Behavior ‐> IntelliCage Plus ‐> Archive.
  b. The data can be displayed in the Analyzer to provide a first overview.
  c. The analysis of the results depends on the research question and can be done, for example, with the statistics program R. Examples of the evaluation of a consumer demand experiment in the IntelliCage with R can be found here: https://zenodo.org/records/6325238

#### Limitations and additional information

The IntelliCage is embedded in a P2000 cage (Tecniplast). This provides a relatively large amount of space for keeping and testing several mice together. When placing animals in the IntelliCage and forming test groups, their age must always be taken into account. Very young mice (3‐4 weeks old) are relatively small, but they increase significantly in body weight and size over time, so stocking density is limited. In each compartment, sufficient nesting material must be provided to enable nest building, as well as shelter in the form of houses offering enough space for more than two or three adult mice. Additionally, consideration should be given to facilitating other natural behaviors, such as climbing, digging, and gnawing. To provide the mice with more space, another cage can be connected to the IntelliCage by drilling holes in the cages to connect them via tunnels.

However, bear in mind that the more shelter options there are, the more difficult daily visual checks become. The IntelliCage itself only allows limited visibility from the outside. Therefore, it must be positioned in the room so that it can ideally be viewed from several sides. It can also take more time to remove the mice than it would with a conventional cage, as the small grid lid cannot easily be removed to allow access to every area.

It has been anecdotally reported that, within the IntelliCage, individual mice can be observed pushing, shoving, and pulling each other out of corners. This could negatively affect the mice's learning performance. However, it has repeatedly been shown that mice kept in groups in the IntelliCage can perform learning tasks (Kahnau et al., 2021; Krackow et al., 2010; Mechan et al., 2009; Voikar et al., 2018), and the system is sensitive enough to detect differences in strain (Endo et al., 2011; Krackow et al., 2010).

In learning tasks that require a higher level of concentration or focus, however, mutual influence could negatively affect the results and make interpreting the data difficult.

For instance, in a consumer demand test, the mice must perform more nosepokes each day to access the drinking bottles (Kahnau et al., 2022a, 2022b). This enables the strength of preference for or aversion to different liquids to be determined. Because the required number of nosepokes takes more time given that the mice must perform more of them, it is important that they can do so undisturbed by group members. The AnimalGate (TSE) allows the IntelliCage to be connected to another cage, enabling individual animals to be entered into the IntelliCage one at a time. This means that only one mouse is in the IntelliCage at a time, while the other group members remain in the second cage (Kahnau et al., 2022a, 2022b; cognitive bias test in the IntelliCage‐based setup: Kahnau et al., 2022c, 2023). This setup makes it possible to obtain individual data from mice kept in groups.

Learning tasks in the IntelliCage use water (or other liquids) as a reward. If the animals do not learn how, where, and/or when they have access to water, water must be provided to them after 24 hr at the latest. This can be achieved by unlocking all corners as correct corners for these animals and/or by removing the time restrictions. Alternatively, water can be offered to the mice outside the IntelliCage in a separate cage. However, repeatedly providing water separately can negatively affect the mice's motivation to learn which corner and pattern provides access to water and when.

The protocols presented here do not describe an explicit study in which each step of introducing a new method is detailed. Instead, they describe the existing IntelliCage system and provide supplementary information not included in the manual or publications. The aim is to help avoid common mistakes and support the planning and implementation of IntelliCage experiments.

Development of the IntelliCage began in 1978, and it has been commercially available since 2003 (Lipp et al., 2023). Various studies have demonstrated that it is possible to investigate the learning behavior of group‐housed mice using this system. For instance, the involvement of the central amygdala in signal transmission for rewarded learning (Knapska et al., 2006), behavioral flexibility (Endo et al., 2011), and stress response (Branchi et al., 2010; Gapp et al., 2014; Nagaeva et al., 2023) have been examined. Various liquids can also be used in the IntelliCage (Bramati et al., 2023; Kahnau et al., 2022a; Pfefferle et al., 2025).

Advantages of the IntelliCage include testing mice in their familiar environment, independently of humans (significantly reducing handling), in social groups and during their natural active phase. In addition, the system runs automatically, eliminating the need for a human operator and reducing direct influence on the animals and saving time. These advantages support the reproducibility of the data.

To obtain information from experiments with real added value, it is imperative that all information about experiments be provided not only in protocols, but also in scientific publications. This includes all information about the animals (e.g., age, sex, strain, and husbandry), as well as the data and codes used for analysis (e.g., Jaap et al., 2024; Habedank et al., 2021; Kahnau et al., 2022a, 2022b, 2022c).

## Basic Protocol 2 IntelliCage CLEANING

This protocol outlines the necessary steps for cleaning an IntelliCage. It is necessary to clean the IntelliCages regularly, both for hygiene and to ensure that the sensors work properly.

The frequency of cleaning depends on the number of mice in the IntelliCage, whether they are males or females (males mark more frequently), and whether another cage is attached. It also depends to a large extent on the experimental design and must be considered before the start of the experiment. For example, if there are 12 males in each IntelliCage with no additional cage attached, it is good practice to clean the IntelliCage weekly.

Attention should also be paid to the timing of cage cleaning, as the whole procedure affects the activity of the mice (Kahnau et al., 2022a, 2022b; Pernold et al., 2019). Therefore, cleaning should preferably be done after a learning module is completed.

### Additional Materials (also see Basic Protocol 1)

Cotton buds
Paper towels
Ethanol
Isopropanol
Water and soap

#### IntelliCage cleaning

**Figure 10:**
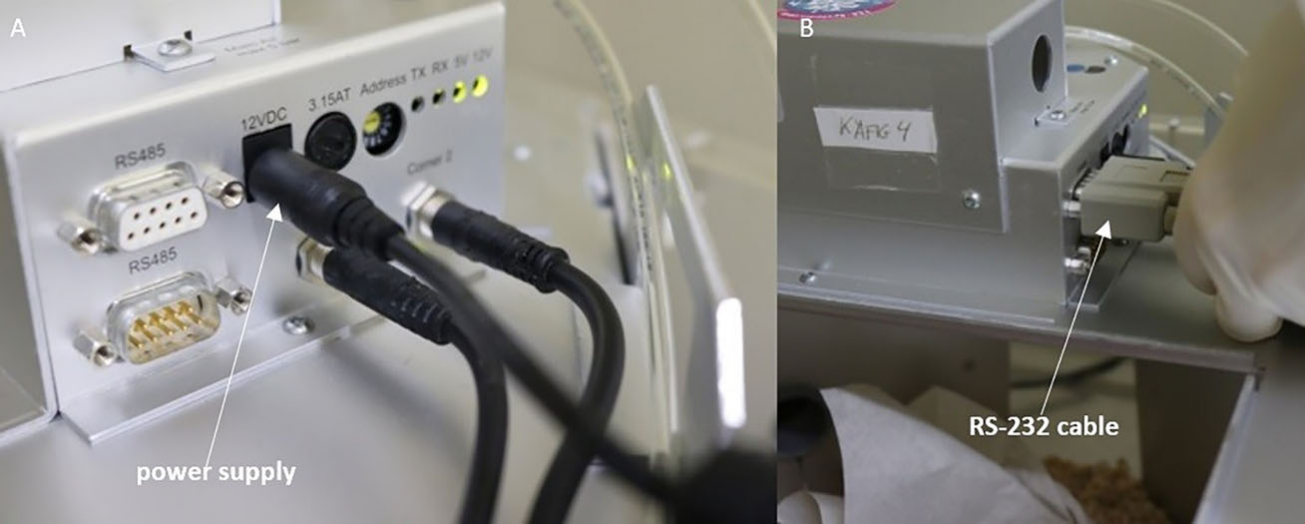
Power supply and RS‐232 cable on the mainboard of the IntelliCage.

1 Stop the IntelliCage in the Controller module of the IntelliCage Plus software:
  a. Click “Console”.
  b. Click “Stop” (on the right).
2 Remove mice from the IntelliCage by handling tube, and place them in a separate cage with at least bedding and nesting material.
  Alternatively, cub handling can be chosen as a stress‐free handling method.
3 Disconnect the power supply (Fig. 10A) and the RS‐232 cable (Fig. 10B).

**Figure 11:**
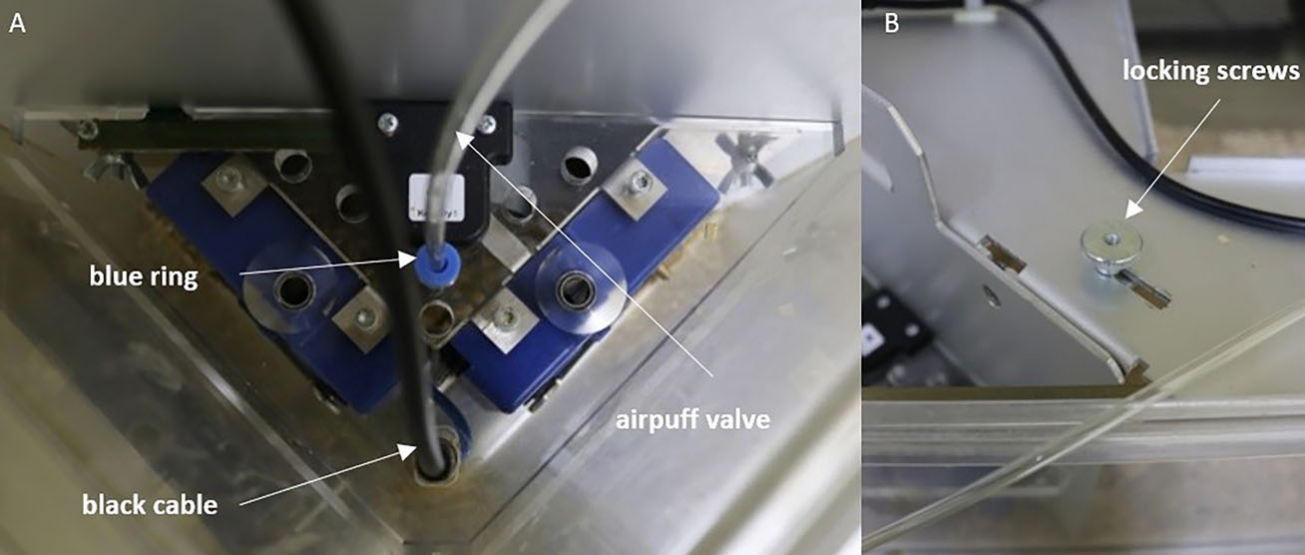
Cable connections to the IntelliCage corner.

4 Carefully remove the water bottles one at a time, removing them vertically upward. If necessary, have a paper towel handy to catch any dripping water. Be careful not to drop any water on the electronics.
*CAUTION*: Some drinking spouts can get stuck in the corner—do not pull on the bottle abruptly, to prevent it from coming off the spout.
5 Remove the cable connections at the corners (Fig. 11A):
  a. Pull the black cable vertically upward and press down with the other hand against the corner.
  b. Remove the airpuff valve by pressing the blue ring down with one hand while simultaneously pulling the valve vertically upward.
    The valves are only needed when working with air puffs, which can be used as a punishment, e.g., when a mouse makes an incorrect nosepoke.

**Figure 12:**
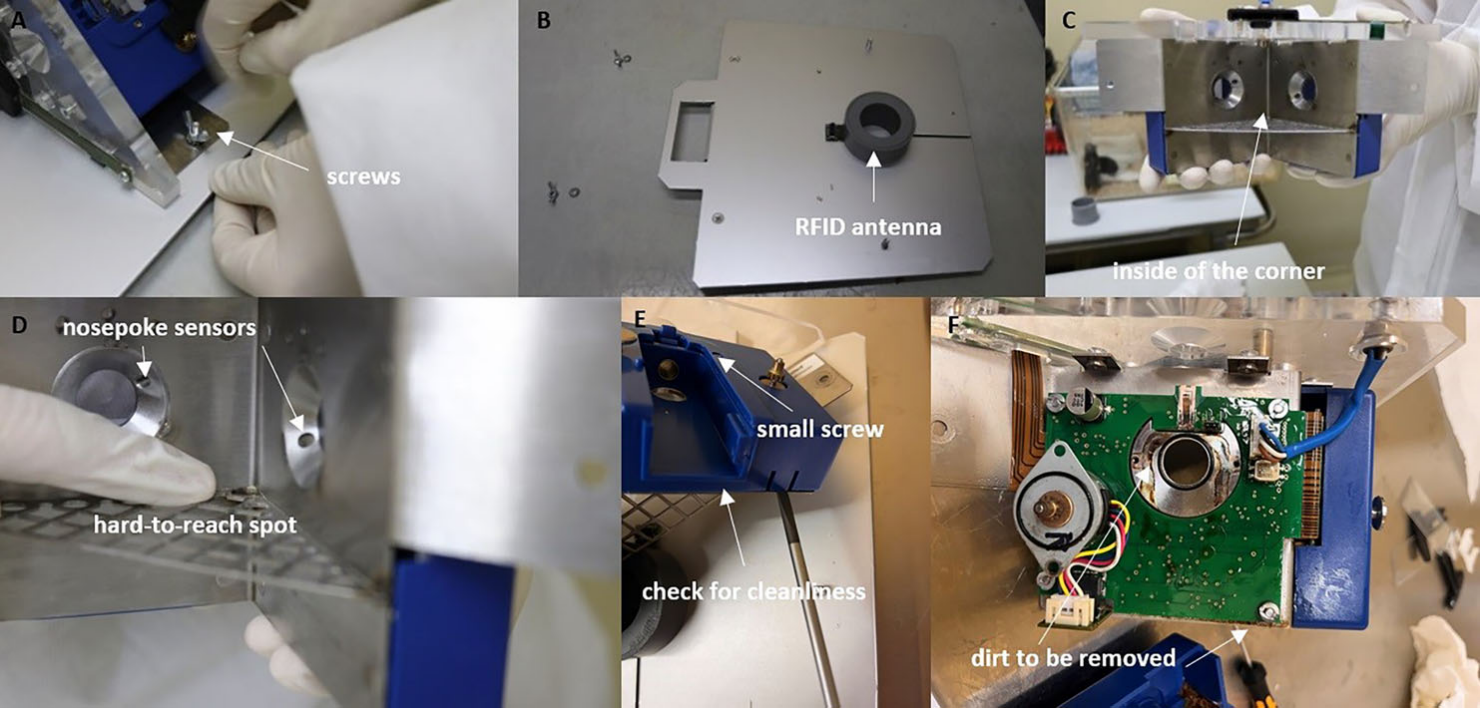
Illustrations of cleaning process.

6 Remove the corners by loosening the locking screws on the metal plate on top and pushing them to the side (Fig. 11B).
7 Loosen the corner of the metal wall to which the RFID antenna is attached by loosening the screw (Fig. 12A).

8 Clean the plate and corner with water, soap. and ethanol (Fig. 12B and C).
*CAUTION*: The electrical parts must not get wet!
9 Clean hard‐to‐reach areas with a cotton bud (Fig. 12D). Check whether there is dirt under the blue shell. If necessary, remove the blue shell by first loosening the small screws and then carefully lifting off the blue shell with a screwdriver (Fig. 12E).
*CAUTION*: Clean the plate only with isopropanol (Fig. 12F).

#### IntelliCage reassembly

10 After cleaning, reassemble the system and prepare for use:
  a. Reassemble everything, taking care **not to mix up any corners** or parts of the corners.
    Mixing up the corners, more precisely the RFID antennas, can lead to faulty functioning of the RFID antennas.
  b. After all corners are correctly positioned again, tighten all screws and attach all cables.
  c. Fill the water bottles to a **maximum of 250 ml** and carefully position them in the corners. Make sure that no water gets onto the electronics.
  d. Connect the power cable and the RS‐232 cable.
    The system is now ready for you to proceed with the experiment as described in Basic Protocol 1, steps 12‐21. However, the habituation step is only necessary when the animals are placed in the IntelliCage for the first time or after a long break.

## COMMENTARY

### Background Information

These protocols provide information on important considerations for planning an IntelliCage experiment, information on conducting a learning experiment, and cleaning instructions. This information is often absent from scientific publications, yet it is crucial, as preventable errors can only be avoided through knowledge. The IntelliCage is an automated home cage based test system that collects individual data from animals kept in groups, with minimal influence from the experimenter. As with all technical equipment, however, experience with the system is very helpful in order to avoid future mistakes.

### Critical Parameters

It is important to check the functionality of the IntelliCage before starting the experiment and after each cleaning. The data should also be checked in the first few days to see if the mice are behaving as expected or if there are errors in the settings in the Designer (e.g., licks only occur in the corners that are set as correct, the day pattern settings are correct, only the first nosepoke opens the door). Adjustments may need to be made in the Designer.

After starting the experiment and after cleaning, the power supply should be checked, especially if you are working with a laptop. If the laptop runs out of power, the IntelliCage will no longer work.

Do not mix up the corners, as these are tuned to each other. The corners could be labeled, for example.

Disable automatic software updates (Windows settings) while running an experiment.

Set a short inactivity timeout to automatically turn off the screen.

Deactivate the standby mode of the operating system software (e.g., Windows).

Mute all audio.

Check that all access points and the food rack are firmly closed to prevent mice from escaping.

Check food and water daily and refill/replace if necessary. *NOTE*: Do not fill water bottles above 250 ml.

It may happen that an individual mouse stuffs nest material into the corners of the IntelliCage. In this case, remove the nest material and consider whether more or different shelter options need to be provided.

### Troubleshooting

The IntelliCage system consists of various parts that only function properly when assembled and used correctly. There is a risk of parts being mixed up, errors occurring when creating an experiment in the Designer or parts breaking. The Troubleshooting Guide (Table 2) is intended to highlight potential problems and offer appropriate solutions.

**Table 2:** Troubleshooting Guide

Problem	Possible cause	Solution
IntelliCage cannot be controlled in the “Controller”.	The wrong COM is specified in the settings.	Set correct COM. If this does not solve the problem, contact TSE.
Presence sensor does not respond (by hand or mouse).	The presence sensor may be contaminated. The experimenter's hands are too cold.	Clean with ethanol or isopropanol. If this does not solve the problem, contact TSE. Warm your hands, e.g., with warm water
Nosepoke sensor does not respond (by hand or mouse).	The nosepoke sensor may be contaminated.	Clean with ethanol or isopropanol. If this does not solve the problem, please contact TSE.
The mouse's transponder number is not registered during a visit.	A typing error was made when entering the transponder number into Designer.	Correct the number in the Designer, then save and reload the experiment.
Lickometer does not respond (by hand or mouse).	Gloves prevent direct contact.	Remove gloves. If this does not solve the problem, please contact TSE.
The RFID antenna is not responding.	The corners or the plate with the ring antenna were not inserted in exactly the same position as when delivered by TSE. The RFID antenna is defective.	Mixing up the corners or antennas can cause interference. Label the corners/antennas upon delivery. If they have been mixed up, swap all the corners/antennas until all the RFID antennas respond correctly again. Contact TSE.
When checking the sensors in the corners, the wrong corners are activated in the Controller.	The cable connections have been mixed up. There is one cable for each corner, running from the mainboard to that corner, which must not be mixed up.	Adjust cable connections.
No mouse has drunk in the past 24 hr.	Error in Designer when assigning clusters, modules, and/or connections of individual events. The water bottles are filled to >250 ml.	Check the Designer document and correct it if required. Fill to a maximum of 250 ml. Otherwise, negative pressure will be created and no water will be able to flow out of the drinking bottle.
An individual mouse has not drunk for the last 24 hr.	A typing error was made in entering the transponder number into Designer. The transponder is defective. The transponder is located too far back on the back of the mouse, rather than in the neck region or the area of the tail root.	Correct the transponder number in Designer. Check the transponder manually using an RFID reader. If the transponder is not recognized manually either, it is probably defective. Exclude the mouse from the experiment or implant a new transponder. However, note that this procedure may affect the animal's behavior. Consider whether it is advisable to perform surgery to correct the transponder's position, as this may affect the animal's behavior. Alternatively, the mouse should be excluded from the experiment.
The laptop has switched off.	The power cable was not connected.	Connect the power cable.

### Statistical Analysis

All data recorded during the experiment—number of visits (correct and incorrect), visit time, number of nosepokes (correct and incorrect), number of licks—are stored on the PC/laptop under Documents ‐> New Behavior ‐> IntelliCage Plus ‐> Archive.

The data can be displayed in the Analyzer and thus provide a first overview.

The analysis of the results depends on the research question and can be done, for example, with the statistics program R. Examples of the evaluation of a consumer demand experiment in the IntelliCage with R can be found here: https://zenodo.org/records/6325238

### Understanding Results

The IntelliCage is used to study the learning behavior of mice living in groups. The animals must learn which corner and which water bottle they have access to at what time. The IntelliCage can be used to create relatively simple learning experiments, such as Place Learning (whereby each mouse is assigned a single corner as the correct corner), or more complex learning tasks in combination with sound (Kahnau et al., 2023). The mice can also be made to “work” for access to different liquids by increasing the number of nosepokes required to open the doors each day, in order to investigate their preferences or aversions to different liquids (Kahnau et al., 2022a, 2022b).

The IntelliCage system records various behaviors. Various sensors record visits (entering a corner), as well as whether nosepokes or licks were made. Based on this data, conclusions can be drawn about learning behavior, preference or aversion.

Depending on the experimental setup, correct or incorrect visits involving nosepokes can be compared between different treatments. It is advisable to evaluate nosepoke visits, as visits alone may not be sufficiently meaningful. Mice may repeatedly enter the corners of the cage simply by running around, without any apparent motivation to drink.

Therefore, it is advisable to define a learning criterion in advance. This criterion may then depend on the experimental design. If only one of the four corners is the correct one, there is a 25% probability of finding it randomly. Therefore, the learning criterion should be set above 25%.

As the protocol presented here is intended to provide a general overview of how to conduct an IntelliCage experiment, and as individual experimental designs can be created, no specific example of evaluation are presented.

### Time Considerations

Although the IntelliCage system is automated, regular checks of the technology are essential (Table 3). To monitor the animals’ learning behavior, it is advisable to check the data promptly, so any design flaws can be rectified immediately. As the animals only have access to water in their corners, it is necessary to check daily whether they have all drunk. If not, they must be given water separately. You should also check for technical faults or whether the learning experiment is too complex.

**Table 3:** IntelliCage Time Management

Task	Time	Task frequency
Design an experiment	Easy, e.g., Place Learning: 20‐30 min Complex, e.g., cognitive bias*^a^*: 60 min	Just once, at the beginning of the experiment.
Load and start an experiment	5 min	Just once, at the beginning of the experiment. Multiple IntelliCages (up to 8) can be controlled via one PC, so the experiment only needs to be loaded and started once and then after each stop (e.g., for cleaning).
Retrieve data	5 min	The experiment can (theoretically) run for weeks without the system having to be stopped. However, the IntelliCage must be cleaned regularly. The IntelliCage should be stopped during cleaning. Normally, you only retrieve the data once the experiment, for example Place Learning, has been completed (e.g., after 1 week). Only then (or during cleaning) is the system stopped, so that you can transfer the data from the PC to a USB stick, for example, and edit/evaluate it elsewhere.
System restart	∼1‐1.5 hr	The system must be restarted after cleaning. The procedure then takes ∼1‐1.5 hr, including cleaning and subsequent testing of individual sensors, such as the nosepoke sensors or lickometers, and the (weekly) health check of the mice. How often the IntelliCage needs to be cleaned depends on how many animals are kept in the IntelliCage (up to 16 mice per IntelliCage are allowed, depending on their body weight), whether they are male or female (males mark significantly more, which is why more frequent cleaning is necessary), and whether another cage is connected. A system restart should be performed when a given experiment is finished and a new one is to be started. How often this happens depends on the individual experiment plan.
Visual check	10 min/day	The daily visual check includes checking whether all mice have drunk water within the past 24 hr. If not, the mouse must be given water separately, which takes 15 min. You should then check that all the sensors are working by looking at the nosepoke numbers and visits, which only takes a few minutes. If an error is detected, it must, of course, be rectified, which can take a considerable amount of time.

^*a*^ Note that the use of this system for cognitive bias testing is not common; I use the IntelliCage in a different way from other researchers.

### Author Contributions

**Pia Kahnau**: Conceptualization; writing—original draft; writing—review and editing; visualization.

### Conflict of Interest

I declare that I have no financial, business, or personal relationships that could be considered a potential conflict of interest in connection with this work.

## Data Availability

Data sharing is not applicable to this article as no data were created or analyzed in this study.
